# Quinalizarin as a potential antifungal drug for the treatment of *Candida albicans* fungal infection in cancer patients

**DOI:** 10.1128/spectrum.03652-23

**Published:** 2024-01-30

**Authors:** Monika Janeczko, Elżbieta Kochanowicz, Kamila Górka, Tomasz Skrzypek

**Affiliations:** 1 Department of Molecular Biology, Faculty of Medicine, The John Paul II Catholic University of Lublin, Lublin, Poland; 2 Department of Biomedicine and Environmental Research, Faculty of Medicine, The John Paul II Catholic University of Lublin, Lublin, Poland; Agroscope, Switzerland

**Keywords:** antifungal activity, anthraquinones, biofilm, *Candida albicans*, *Candida auris*, cancer, fungal infection, hyphae, SEM, ROS, quinalizarin

## Abstract

This study aims to analyze the antifungal properties of quinalizarin, a plant-derived compound with proven anticancer effects. Quinalizarin exhibited antifungal activity against opportunistic pathogenic *Candida* species and *Geotrichum capitatum*. The treatment with this anthraquinone reduced hyphal growth, inhibited biofilm formation, and damaged mature *Candida albicans* biofilms. Real-time RT-PCR revealed that quinalizarin downregulated the expression of hyphae-related and biofilm-specific genes. The flow cytometry method used in the study showed that both apoptosis and necrosis were the physiological mechanisms of quinalizarin-induced *C. albicans* cell death, depending on the dose of the antifungal agent. A further study revealed an increase in the levels of intracellular reactive oxygen species and alterations in mitochondrial membrane potential after treatment with quinalizarin. Finally, quinalizarin was found to have low toxicity in a hemolytic test using human erythrocytes. In conclusion, we have identified quinalizarin as a potential antifungal compound.

**IMPORTANCE:**

This article is a study to determine the antifungal activity of quinalizarin (1,2,5,8-tetrahydroxyanthraquinone). Quinalizarin has potential antitumor properties and is effective in different types of tumor cells. The aim of the present study was to prove that quinalizarin can be used simultaneously in the treatment of cancer and in the treatment of intercurrent fungal infections. Quinalizarin was identified as a novel antifungal compound with low toxicity. These results may contribute to the development of a new drug with dual activity in the treatment of cancer-associated candidiasis.

## INTRODUCTION

Cancer is one of the most serious problems and one of the most important health challenges worldwide. The number of cancer cases has increased significantly and is estimated at up to 18.1 million new cases and 9.6 million cancer deaths (1, 2). There is currently increasing concern about the relationship between microbial infections and cancer. Many studies support the presence of such association, primarily with bacteria, viruses, and fungi as the causal agents. The association of cancer with microbiological infections is a consequence of the transient immunodeficiency that is commonly seen in cancer patients, mainly due to chemotherapy and prolonged neutropenia. The human immune system normally maintains a balance with the body’s microbiome and is capable of fighting off most microbial invasions. In contrast, in immunosuppressed conditions resulting from cancer treatment, these functions are compromised and cancer patients are at increased risk of infections (3, 4).

Invasive fungal infections (IFIs) are one of the most detrimental infectious diseases for patients with cancer. These diseases are the main cause of morbidity and mortality among highly immunocompromised patients with cancer and prolonged neutropenia due to anti-cancer treatment. The epidemiology of IFIs in immunocompromised cancer patients is mainly associated with *Candida* infections. It is notable that as many as 35% of candidemias are diagnosed in patients with hematological malignancies or solid tumors as the underlying disease (5). These opportunistic pathogens are the most common cause of superficial vaginal or mucosal oral infections, but they may also enter the bloodstream, leading to deep tissue infections (6). The penetration of these fungal species into the bloodstream and their hematogenous dissemination, causing candidemia, can be life-threatening, especially in immunocompromised patients and those hospitalized with hemato-oncological malignancies. The candidemia mortality rate varies between 30% and 60%. Cancer patients often suffer from chronic disseminated candidiasis (hepatosplenic candidiasis), which is a complication of highly toxic chemotherapy. In neutropenia, internal organs , such as liver, spleen, kidneys, lungs, as well as skin and bone, become seeded by *Candida* in the bloodstream. Importantly, it has been proved that neutropenia, a high APACHE score, and disseminated candidiasis are associated with poorer outcomes (7).

Relatively few studies have analyzed the potential influence of fungal diseases on tumor establishment and progression (8–11). *Candida* can promote cancer by several mechanisms: production of carcinogens, such as acetaldehyde, which can conduce to the development of the disease, induction of an inflammatory process that may favor metastatic progression, molecular mimicry, and the Th17 response of the immune system (12).

At least 15 distinct *Candida* spp. can cause human disease, but the majority of invasive infections, also those associated with cancer, are caused by five pathogens*: Candida albicans, Candida glabrata, Candida tropicalis, Candida parapsilosis*, and *Candida krusei* (13–16). In certain parts of the world, a previously rare organism, *Candida auris*, has emerged as a major pathogen (17, 18). The risk factors for *C. auris* infections include cancer and chemotherapy (19). Compared to common *Candida* infections, invasive *Geotrichum capitatum* infections are rare and have been reported exclusively in immunocompromised patients, especially in hematological malignancies and severe neutropenia (20). However, the prognosis is poor with a mortality rate of approximately 50%–75% (21). The most pervasive and problematic cause of infections of all *Candida* species is *Candida albicans—*part of the commensal microbiota of more than half of the healthy population. The detection rate of *C. albicans* is 70%–90% among all candidiasis-causing fungi (22). The mortality rate of *C. albicans-*infected patients is up to 43.6% due to candidemia (23).

The IFI predisposing factors include the interrelated elements of fungal virulence traits, antifungal drug resistance or tolerance, and biofilm formation. *C. albicans* pathogenicity is facilitated by a number of virulence factors and fitness attributes, the most important of which are the adherence to host tissues and medical devices, morphological transition between yeast and hyphal forms, biofilm formation, and secretion of hydrolytic enzymes (22). Virulence factors facilitate target adherence, host defense evasion, tissue damage, thermotolerance, and adaptation to unfavorable microenvironments, including hypoxia and iron-poor conditions. Moreover, these traits block out exposure to antifungal drugs and reduce their effectiveness. Most importantly, the vast majority of *C. albicans* infections are associated with biofilm formation (23). Moreover, biofilm is a particular iatrogenic and treatment-related factor that may be the most prevalent cause of IFIs. *Candida* biofilms are complex three-dimensional communities that often incorporate bacterial pathogens. Biofilms often form on indwelling medical devices, other implanted foreign bodies, or mucosal surfaces. These forms of growth cause a spectrum of clinical entities ranging from catheter-related fungemia to endocarditis and vaginal infections. Biofilms formed by *C. albicans* are an increasingly recognized clinical problem, and most antifungal agents show limited penetration ability and weak activity against biofilms. Due to the high health burden on cancer patients associated with fungal disease, the treatment of these infections needs to be potent and effective (24).

Here, we present, for the first time, the influence of the anti-cancer drug quinalizarin on pathogenic yeasts, *Candida* species and *G. capitatum* in relation to the possibility of using this drug in a simultaneous treatment of cancer and fungal infections. Quinalizarin (1,2,5,8-tetrahydroxyanthraquinone) is a compound extracted from the roots of Chinese herbal medicinal *Rubiaceae* plants or can be artificially synthesized (25). Its anthraquinone ring is similar to the nuclei of antitumor drugs such as doxorubicin and daunorubicin (26). Quinalizarin has potential antitumor properties through its influence on cell proliferation, metastasis, and apoptosis. Many studies report that quinalizarin is effective in different types of tumor cells (breast cancer, prostate cancer, leukemia T cells, liver cancer, lung cancer, colorectal cancer, and gastric cancer) and angiogenesis (27–35). Other studies also suggest that quinalizarin can increase tumor sensitivity in tumor radiotherapy (36). The anticancer activity of quinalizarin is associated with various mechanisms of action, including modulation of the Akt, MAPK, STAT3, and p53 signaling pathways (32, 34, 35). Furthermore, other reports indicate the compound as a potent and selective inhibitor of protein kinase CK2 (26, 27). In turn, the latest research shows that the modified quinalizarin as Cu^II^ complex shows a remarkable ability to disrupt the activities of human DNA topoisomerase I and II and has anticancer properties. The Cu^II^-quinalizarin complex increases cellular uptake and more effectively binds DNA compared to quinalizarin. On the other hand, the complex, unlike quinalizarin, does not catalyze the flow of electrons from NADH to O_2_ to the extent known from quinalizarin (33).

## MATERIALS AND METHODS

### Microorganisms and culture medium

Eight strains of yeast were obtained from the American Type Culture Collection (ATCC): *C. albicans* ATCC 10231, *C. glabrata* ATCC 15126, *C. parapsilosis* ATCC 22099, *C. tropicalis* ATCC 13803, *C. krusei* ATCC 14243, *Candida kefyr* ATCC 204093, *C. auris* ATCC MYA-5001, and *G. capitatum* ATCC 201230. The clinical isolates of *C. albicans* (CA1-CA20) were obtained as stock cultures from the Jan Boży Independent Public Provincial Hospital in Lublin, Poland. The strains were identified using VITEK 2 YST IC CARDS (Biomerieux). Stock cultures were stored at −70°C, subcultured on YPDA medium (1% yeast extract, 2% peptone, 2% dextrose, and 2% agar), and stored at 4°C. The fungal strain cultures were routinely maintained in YPD medium (1% yeast extract, 2% peptone, 2% dextrose) at 30°C.

### Antifungal agents

Quinalizarin was purchased from Sigma-Aldrich (Sigma-Aldrich, USA), dissolved to a concentration of 5,200 µg/mL in dimethyl sulfoxide (DMSO), and stored at −20°C. Fluconazole was purchased from Sigma-Aldrich; the stock solution was prepared by dissolution in sterile distilled water to a concentration of 6,400 µg/mL and stored at −20°C. The final concentration of DMSO was 1% in all assays.

### Antifungal activity

Minimal inhibitory concentrations (MICs) were determined using the microdilution method described in the guidelines of the Clinical and Laboratory Standards Institute (CLSI) (37). Serial twofold dilutions were prepared exactly as described in the CLSI document. The spectrophotometric method of inoculum preparation, an inoculum concentration of 1.5 × 10^3^ cells/mL, and RPMI 1640 (Sigma-Aldrich, USA) buffered to pH 7.0 with MOPS (Sigma-Aldrich, USA) at 0.165 mol/L were used. A total of 0.1 mL of yeast inoculum was added to each well of the microdilution plates. Drug-free and yeast-free controls were included. The plates were incubated at 37°C for 24 h. The MIC was taken as the lowest concentration of the tested compound that inhibits visible microbial growth. The experiments were performed three times with three replicate wells for each experiment.

Minimal fungicidal concentrations (MFCs) of quinalizarin against reference yeast strains were determined in the broth dilution MIC test by subculturing on Sabouraud dextrose agar plates that did not contain the compound at concentrations corresponding to MIC, 2× MIC, and 4× MIC. For this purpose, 10 µL of the suspension from appropriate wells was applied to agar plates and incubated at 37°C for 48 h. The MFC was defined as the lowest concentration of the agent inhibiting visible growth on a solid medium.

### Formation of hyphae

The effect of quinalizarin as a control on the hyphal growth of *C. albicans* cells was evaluated using both liquid and solid RPMI 1640 medium containing 10% fetal bovine serum (FBS). Yeast cells were grown overnight at 37°C in Sabouraud dextrose broth medium in a shaker at 180 rpm. At the late exponential growth phase, the yeast cells were harvested, washed twice with phosphate-buffered saline (PBS), pH 7.2, and resuspended in PBS to 1 × 10^7^ cells/mL. One hundred microliters of the suspension was used for the assays. *Candida* cells were grown on RPMI 1640 agar medium plates supplemented with or without quinalizarin at 2 and 4 µg/mL. The plates were incubated at 37°C for 24 h. The representative *Candida* colonies were compared with the controls (media without the antifungal agent). The morphology of *Candida* colonies was inspected under a light inverted microscope and imaged using a digital camera. The colonies with hyphae were counted and expressed as a percentage of 100 *Candida* colonies.

In order to visualize the effect of quinalizarin on *C. albicans* cells growing in the liquid media, the yeasts were incubated with 2 and 4 µg/mL of the compound at 37°C for 10 h in a shaking incubator. The liquid *Candida* culture samples (10 mL each) were centrifuged and then resuspended in PBS. The cell suspensions (1 × 10^4^ cells/mL) were gently applied onto microscope slides. The cells were stained with methylene blue and visualized using light microscopy. Evaluation of the inhibition of the yeast-to-hyphal-form transition was accomplished by observation of the number of individual budded cells versus the number of hyphae in the population, as previously described (38). Over 100 cells were counted for each well in duplicate. All assays were repeated three times. The hyphae were expressed as a percentage of 100 *Candida* cells calculated as follows: % hyphae = (hyphal cells/100 cells) × 100%.

### Inhibition of biofilm development

The *C. albicans* cells were grown as biofilms using polystyrene flat-bottomed microtiter plates. The cell suspensions were prepared in the RPMI 1640 medium at a cell density of 2 × 10^6^ cells/mL and dispensed into the wells of microtiter plates (100 µL per well) (39). The effect of quinalizarin on the biofilm formation ability was tested in the presence of 100 µL of different concentrations of the compound (8, 16, and 80 µg/mL). In total, 100 µL of the RPMI 1640 medium containing 1% DMSO without the tested compounds was the negative control. The plates were incubated for 24 h at 37°C. At the end of incubation, the medium was aspirated from the wells and planktonic-phase cells were removed by washing the biofilms three times with PBS pH 7.4). The resulting biofilm was treated with 40 µL of 3-(4,5-dimethyl-2-thiazolyl)-2,5-diphenyl-2H-tetrazolium bromide (MTT) (1 mg/mL), 2 µL of 0.4 mM menadione, and 158 µL of PBS in order to determine its viability. After incubation at 37°C for 3 h, absorbance at 490 nm was measured using a microplate reader (Biotek Synergy HT) (40). The percentage of viability inhibition was calculated based on the values of the experimental group and the growth control. The same *C. albicans* culture procedure was used to evaluate biofilm biomass loss under quinalizarin pressure. After incubation, the biofilms were dried, and their biomass was determined using the crystal violet test (41, 42). The reduction of the biofilm was calculated as a percentage of biomass loss based on the values obtained in the experimental group and the growth control.

The morphology of the *C. albicans* biofilm was inspected under a light microscope and imaged using a digital camera (Delta Optical). In order to visualize the effect of quinalizarin on *Candida* biofilm formation, the microscope slides were immersed in liquid cell culture in RPMI 1640 supplemented with 10% FBS. The initial cell density was set at 2 × 10^6^ cells/mL. Quinalizarin was added at 8, 16, and 80 µg/mL for incubation. An anthraquinone-free culture was the control of the experiment. The samples were exposed to quinalizarin for 24 h at 37°C without shaking. Then, the microscope slides were removed from the liquid culture and rinsed with a saline solution to remove planktonic cells. The biofilms were stained with crystal violet and subjected to microscopic observations.

### Mature biofilm eradication

For preformed biofilms, the *C. albicans* cell suspensions were prepared in RPMI 1640 at a cell density of 1 × 10^6^ cells/mL, and 100 µL of the cell suspensions was dispensed into the wells and incubated at 37°C for 24 h. After incubation, non-adherent cells were gently removed and the wells were washed three times with PBS and filled with 100 µL of twofold dilutions of quinalizarin in RPMI 1640 corresponding to MIC, 2× MIC, and 10× MIC (8, 16, and 80 µg/mL). For the control, 100 µL of RPMI 1640 medium containing a final 1% DMSO concentration was added into selected wells with biofilms. Next, the microtite plates were incubated at 37°C for 24 h. After incubation in these conditions, the medium was aspirated from the wells and nonadherent cells were removed by washing the biofilms as described previously. The MTT assay and crystal violet assay were performed according to the method cited above.

### Scanning electron microscopy

*C. albicans* ATCC 10231 biofilms were grown on poly-L-lysine (PLL)-coated glass. Clean glass coverslips were immersed in a 0.1% PLL solution for 10 min and air-dried overnight, rinsed with sterile deionized water, and air-dried. The cultures were grown overnight in YPD medium and then diluted to an optical density of 1 × 10^6^ cells/mL in RPMI 1640 with 10% FBS. Subsequently, 20 mL of the culture was transferred to sterile Petri dishes containing quinalizarin at 8, 16, and 80 µg/mL or 1% DMSO (control). The samples were exposed to the antifungal for 24 h at 37°C without shaking. The glass coverslips were removed from the Petri dishes and rinsed with sterile water to remove planktonic cells. Then, the samples were rinsed for 2–3 min with a saline solution and fixed in 4%, vol/vol buffered formalin for 1 h. After fixation, the probes were washed in water and dehydrated in a series of alcohol: 30%, 50%, 70%, 90%, and finally in absolute ethanol. The samples were dried in a critical point dryer (Polaron Range, CPD 7501). After this process, the samples were sputter coated (Polaron Range, Sputter Coater 7620) with a 20 nm layer of gold. They were examined using a LEO 1430VP scanning electron microscope (Zeiss, Germany) at an accelerating voltage of 15 kV, with an average working distance of 8–9 mm.

### Relative quantification by real-time reverse transcriptase-PCR

The expression of *HWP1, SAP4, ALS1, ALS3, HYR1, BCR1, EFG1, ECE1,* and *CPH1* genes was evaluated using real-time PCR after treatment with quinalizarin. The *C. albicans* cells were exposed to anthraquinone at the concentration of MIC/2 (4 µg/mL) or 1% DMSO (control) during propagation in liquid culture in Spider medium with 10% fetal bovine serum. After 10-h incubation at 37°C, the yeast cells were collected, and total RNA was extracted with the YeaStar RNA kit (Zymo Research, USA) according to the manufacturer’s instructions. cDNA was then synthesized using a Smart First Strand cDNA Synthesis Kit (EurX, Poland) following the manufacturer’s instructions. For PCR detection of transcripts, we used TaqMan gene expression assays (Lot: 170255, designed by the manufacturer, ThermoFisher Scientific, England) and the Fast Probe qPCR Master Mix (EurX, Poland). The cDNA samples were pre-treated with uracil-N-glycosylase at 37°C for 2 min to degrade any dUMP-containing PCR products and then subjected to initial denaturation at 95°C for 3 min, followed by 40 amplification cycles with denaturation at 95°C for 10 s and annealing/extension at 60°C for 30 s using QuantStudio3 (Applied Biosystems). The relative level of expression of the analyzed genes was calculated with the 2-(∆∆Ct) method using *ACT1* as a reference gene (43).

### Annexin V and PI staining

*C. albicans* cells (1 × 10^8^ cells/mL) exposed to quinalizarin at 4, 8, and 16 µg/mL for 5 h were washed in phosphate-buffered saline and incubated at 30°C for 20 min in 0.02 mg/mL Zymolyase 20T in 0.1 M potassium phosphate buffer (50 mM K_2_HPO_4_, 5 mM EDTA, 50 mM dithiothreitol, 50 mM KH_2_PO_4_, and 40 mM 2-merkaptoethanol) with sorbitol at a final concentration of 1 M and at pH 7.0. Thereafter, spheroplasts were resuspended in 500 µL of 1× binding buffer (BD Pharmingen, kit component) with 1 M sorbitol at a concentration of 1 × 10^6^ cells/mL. Afterward, 100 µL of the cell suspension was transferred to a 5 mL culture tube and the cells were stained with 5 µL of FITC Annexin V and 5 µL of PI. Then, the cells were incubated in the absence of light for 15 min at room temperature and examined by flow cytometry (BD FACSCalibur flow cytometer).

### ROS determination

The effect of quinalizarin on the reactive oxygen species (ROS) formation ability was tested at the concentrations of 2, 4, and 16 µg/mL. Intracellular superoxide formation was detected using CM-H2DCFDA, i.e., the General Oxidative Stress Indicator (ThermoFisher Scientific, England). Overnight cultures were refreshed in YPD medium and incubated with shaking at 30°C until the cell density reached 1 × 10^7^ cells/mL. Subsequently, the *C. albicans* cells were exposed to quinalizarin for 5 h at 30°C with shaking. After incubation, the cells were removed from the growth media via centrifugation at 5,000 rpm for 10 min. Then, the cells were resuspended in PBS buffer containing the probe to provide a final working concentration of 10 µM dye (ROS indicator) and incubated at 30°C for 60 min. After removing the loading buffer, the cells were returned to the prewarmed growth medium and incubated at the optimal temperature for 30 min. The baseline fluorescence intensity of a sample of the loaded cells prior to the exposure thereof to the experimental factors was determined with excitation and emission fluorescence at approximately 492–495/517–527 nm. To prepare a positive control, oxidative activity was stimulated with H_2_O_2_ at a concentration of 100 mM. The experiments were performed in triplicate.

### Mitochondrial membrane potential

The change in the mitochondrial membrane potential (MMP) of *C. albicans* cells was analyzed using JC-10 (analog of JC-1, 5,5′,6,6′-tetrachloro-1,1′,3,3′-tetraethyl-benzimidazolylcarbocyanine iodide). For this purpose, the Mitochondrial Membrane Potential Kit (Sigma-Aldrich, USA) was used. The cells treated with quinalizarin at 2, 4, and 16 µg/mL for 5 h were washed and stained with JC-10 for 30 min in the dark. The red fluorescence intensity (*λ*_ex_ = 540 nm/*λ*_em_ = 590 nm) and green fluorescence intensity (*λ*_ex_ = 490 nm/*λ*_em_ = 525 nm) were monitored using a microplate reader. The ratio of red/green fluorescence intensity was used to determine MMP. An untreated sample and a 50 µM carbonyl cyanide 4-(trifluoromethoxy)phenylhydrazone (FCCP)-treated (Sigma-Aldrich, USA) sample were used as negative and positive controls, respectively.

### DNA damage and nuclear fragmentation

Cells with reduced amounts of DNA due to its fragmentation during apoptosis were identified after cell fixation and 4′,6-diamidino-2-phenylindole dihydrochloride (DAPI) staining. Cells were incubated with 2, 4, and 16 µg/mL of quinalizarin for 5 h and untreated control cells were fixed for 1 h 30 min at 4°C with a 0.1% sterile NaCl water solution containing 2.5% formaldehyde and stained for 20 min by adding 4 µg/mL DAPI (Sigma-Aldrich, USA). The quinalizarin-untreated cells (control) were incubated and fixed in the same manner. The excess stain was removed by threefold centrifugation for 5 min at 5,000 rpm, and the cells were washed with 0.1% sterile NaCl water solution. The DAPI complexed with fungal DNA was then solubilized in 95% ethanol for 15 min under gentle shaking. Fluorescence was measured at 350 nm excitation and 510 nm emission wavelengths using a microplate fluorescence reader (Biotek Synergy HT).

To visualize DNA fragmentation, genomic DNA isolated from the *C. albicans* cells was analyzed by agarose gel electrophoresis. After incubation with quinalizarin at 0, 2, 4, and 16 µg/mL for 5 h, the cells were centrifuged and genomic DNA was isolated using the DNA Mini Kit (Syngen Biotech, Poland). The samples were loaded onto 1% agarose gel in the presence of propidium iodide. Electrophoresis was carried out for 30 min at a constant voltage of 100 mV; then, the gel was photographed.

### Hemolytic assay

Human erythrocytes were harvested by 10-min centrifugation at 2,000 rpm at 4°C. PBS was added to the pellet to yield 10% (vol/vol) erythrocytes in PBS suspension and centrifuged as previously. The washing procedure was repeated until a transparent supernatant was obtained. After washing, the erythrocytes were finally resuspended in PBS buffer at a final concentration of 2%. Simultaneously, appropriate concentrations (in the range of 0.8–160 µg/mL) of quinalizarin were prepared in a final volume of 50 µL DMSO. Total hemolysis was achieved with 1% Triton X-100. The compound prepared in this way was mixed with 450 µL of the 2% erythrocyte suspension and incubated for 1 h at 37°C. Next, the samples were centrifuged at 2,000 rpm for 10 min at 4°C. One hundred fifty microliters of the supernatant fluids were transferred to a flat-bottomed microtiter plate, and the absorbance was measured spectrophotometrically at 450 nm. The percentage of hemolysis was calculated with the following formula: [(A_450_ of quinalizarin-treated sample) – (A_450_ of buffer-treated sample)/(A_450_ of Triton X-100-treated sample) – (A_450_ of buffer-treated sample)] × 100%.

### Statistical analysis

All data are expressed as a mean ± SD (standard deviation) of three independent experiments. Statistical significance between the treated and control groups was analyzed by Student’s *t*-test using GraphPad Software version 9.1.1 (San Diego, CA, USA). Differences between the samples treated with quinalizarin at various concentrations and untreated controls were considered to be significant when *P* < 0.05.

## RESULTS

### Anti-*Candida* activity

Our main goal in this study was to determine the potential antifungal properties of quinalizarin and elucidate the mechanisms of its activity. The microdilution assay used for the determination of the MIC revealed that quinalizarin inhibited the growth of all the tested strains: *C. albicans*, *C. glabrata, C. parapsilosis, C. tropicalis, C. krusei, C. kefyr, C. auris,* and *G. capitatum* with the MIC values of 0.5–128 µg/mL (Table 1). It seems particularly important that quinalizarin is effective against clinical isolates exhibiting resistance to fluconazole (strains numbered CA1-10) because the emergence of resistance to the available classes of antifungal agents, including fluconazole, is a major problem limiting successful patient outcomes. Additionally, the fungicidal properties of quinalizarin were assessed by determining the MFC. The MFC was defined as the lowest drug concentration that resulted in 100% inhibition of growth of the organism. An antifungal agent was deemed fungicidal *a priori* if the ratio of the MFC to MIC did not exceed 4. An antifungal agent with a ratio higher than four was considered fungistatic (44). We found that the MFC/MIC ratios for all microorganisms were higher than 4, indicating that quinalizarin was fungistatic.

**TABLE 1:** MIC values (µg/mL) of quinalizarin and fluconazole^*a*^

Yeast species	MIC (µg/ml)	
	Quinalizarin	Fluconazole
*C. albicans* ATCC 10231	8	64
*C. glabrata* ATCC 15126	32	64
*C. parapsilosis* ATCC 22099	128	1
*C. tropicalis* ATCC 13803	128	64
*C. krusei* ATCC 14243	32	64
*C. kefyr* ATCC 204093	64	0.5
*C. auris* ATCC MYA-5001	64	16
*G. capitatum* ATCC 201230	64	>64
CA1	2	64
CA2	64	>64
CA3	16	>64
CA4	8	>64
CA5	8	>64
CA6	16	>64
CA7	16	64
CA8	8	64
CA9	0.5	64
CA10	8	>64
CA11	16	32
CA12	8	16
CA13	16	16
CA14	8	32
CA15	8	8
CA16	16	8
CA17	0.5	4
CA18	8	4
CA19	16	32
CA20	8	8

^*a*^ Clinical *C. albicans* isolates are numbered CA1–20.

Further experiments were conducted using *Candida albicans* as the most common representative species among pathogenic yeasts.

### Inhibition of hyphae formation

**Fig 1:**
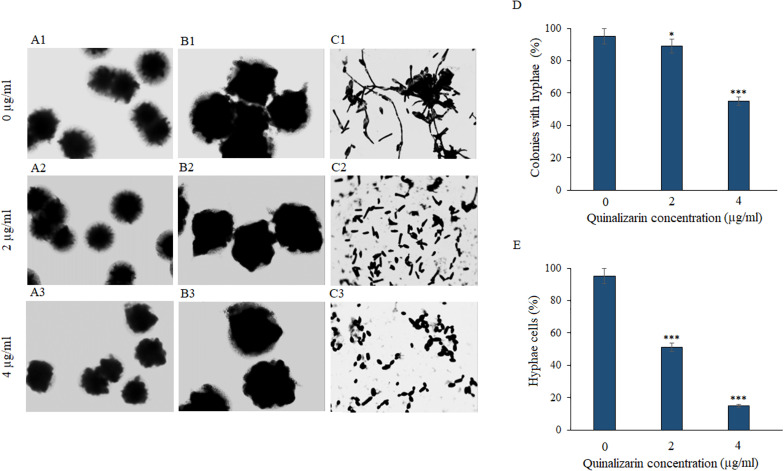
Microscopic images of whole *C. albicans* ATCC 10231 colonies in solid RPMI 1640 medium supplemented with 10% FBS and quinalizarin at 2 and 4 µg/mL (A1–A3, magnification 40×; B1–B3, magnification 200×); microscopic images of *C. albicans* cells in liquid RPMI 1640 medium supplemented with 10% FBS and quinalizarin at 2 and 4 µg/mL (C1–C3, magnification 1,000×); reduction of the number of hyphae-forming colonies (**D**) and hyphae-forming cells (**E**) induced by quinalizarin at 2 and 4 µg/mL. Data are expressed as mean ± standard error of three independent experiments. *P < 0.05; **P < 0.01; and ***P < 0.001 [significance compared to the control (untreated cells) set at 100%].

The yeast-to-hyphae transition is a critical phenomenon of fungal biofilm formation and an important virulence factor. Hyphal morphogenesis can be induced by multiple stimuli, including special media (45). To verify the effect of quinalizarin on *C. albicans* hyphae, the fungi were grown in hyphae-inducing liquid and solid RPMI 1640 medium in the presence of various concentrations of the compound (2 and 4 µg/mL). The light microscope images showed that the increase in the concentration of the compound was accompanied by a decrease in the number of hyphae in the liquid culture and in the culture on the solid medium, where whole colonies of the fungus were observed. As shown in Fig. 1, quinalizarin at the concentration of 4 µg/mL inhibited the formation of hyphae around *Candida* colonies growing on a solid medium (Fig. 1A3 and B3), while this effect at the concentration 2 µg/mL was practically indiscernible (Fig. 1A2 and B2). In contrast, the drug-free control culture showed colonies with distinct hyphae at the edges (Fig. 1A1 and B1). Similarly, in the control liquid culture (without quinalizarin), there were numerous hyphae entangled in cell aggregates (Fig. 1C1). In turn, a significant loss of hyphae and their separation were visible in the drug-supplemented cultures (Fig. 1C2 and C3).

The quantitative analysis of the colonies with hyphae in relation to the smooth colonies grown on solid RPMI 1640 medium showed a significant loss of colonies with hyphae (up to 56% compared to the untreated control culture) induced by quinalizarin at a concentration of 4 µg/mL (Fig. 1D). In turn, the quantification of hyphal cells and yeast cells in the liquid culture in RPMI 1640 showed a significant reduction of hyphae to 18% already at the quinalizarin concentration of 4 µg/mL (Fig. 1E).

### Inhibition of biofilm formation and destruction of mature biofilm

**Fig 2:**
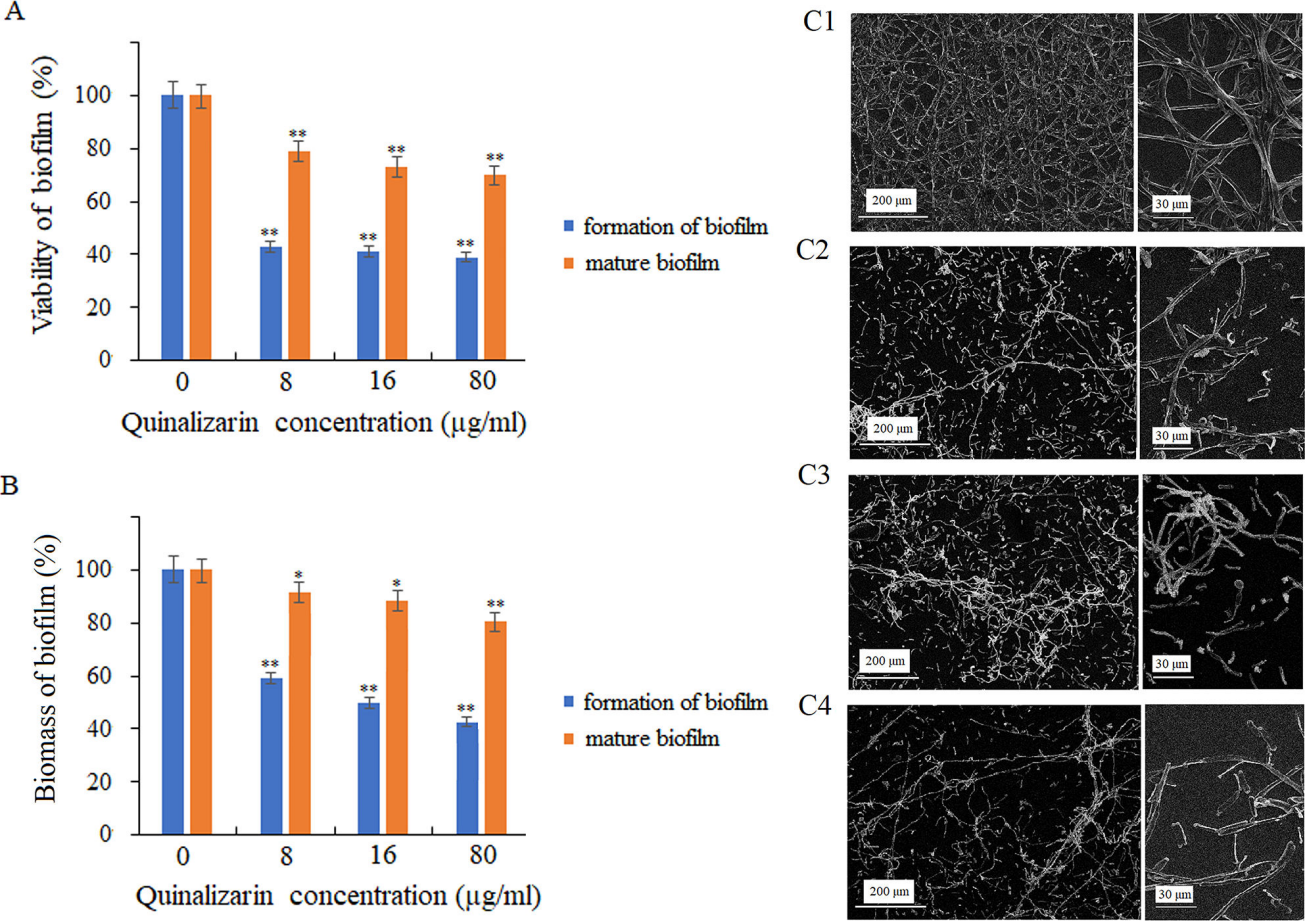
Effect of quinalizarin on biofilm formation by *C. albicans* and reduction of mature biofilm metabolic activity (**A**) and biomass (**B**). Representative scanning microscopy images of *C. albicans* biofilm, control (untreated) cells (**C1**); treatment with quinalizarin at 8 µg/mL (**C2**), 16 µg/mL (**C3**), and 80 µg/mL (**C4**). Data are expressed as mean ± standard error of three independent experiments. *P < 0.05; **P < 0.01; and ***P < 0.001 [significance compared to the control (untreated cells) set at 100%].

In this study, it was proved that quinalizarin reduced the viability and biomass of *C. albicans* biofilm during biofilm formation for 24 h at 37°C. The application of quinalizarin at concentrations of 8–80 μg/mL showed that the compound reduced the viability of the fungal cells to 43%–39% (Fig. 2A). In addition, quinalizarin at 80 µg/mL led to up to 46% reduction in the biomass of the forming biofilm, compared to the control (untreated cells) (Fig. 2B).

The biofilm development follows sequential steps over a period of 24–48 h (15). Interestingly, our findings demonstrate that quinalizarin applied to the biofilm at the concentration corresponding to the MIC value (8 µg/mL) reduced the metabolic activity of the cells to 79%, compared to the control (biofilm without quinalizarin). The increase in the concentration of the compound to 16 and 80 µg/mL resulted in a reduction of the mature biofilm viability to 73% and 70%, respectively (Fig. 2A). Moreover, the treatment with quinalizarin also resulted in a reduction in mature biofilm biomass, as shown in Fig. 2B.

Additionally, morphological changes in the mature *C. albicans* biofilm in the presence of quinalizarin at 8, 16, and 80 µg/mL were visualized by the SEM analysis. The untreated cells (control) were able to form mature biofilms with dense hyphal structures surrounding the yeast cells. In turn, a lower density was observed in the scaffold in the presence of quinalizarin (8, 16, and 80 µg/mL) after 24 h of culture, compared to the untreated culture (Fig. 2C1 through C4).

### Analysis of the expression of biofilm-related genes

**Fig 3:**
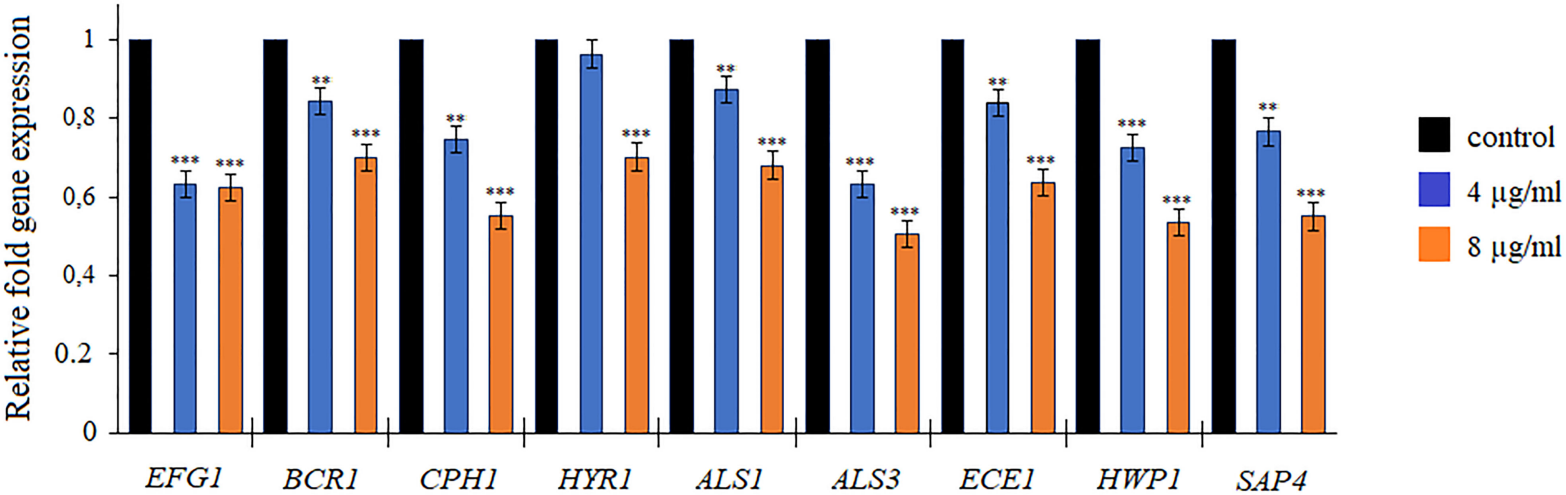
Relative expression of hyphae-related and biofilm-related genes detected by qreal-time PCR. Quinalizarin was used at 4 and 8 µg/mL. The level of gene expression was displayed after normalization with the internal control housekeeping gene ACT1. Data are expressed as mean ± standard error of three independent experiments. **P < 0.01 and ***P < 0.001 [significance compared to the control (untreated cells) set at 1.0].

To further investigate the effects of quinalizarin on *C. albicans* biofilm development, the expression of the known hypha-related and biofilm-related genes, *EFG1, BCR1, CPH1, HYR1, ALS1*, *ALS3, ECE1, HWP1,* and *SAP4,* was analyzed by qRT-PCR. The data in Fig. 3 indicate that the genes were downregulated after the exposure of *C. albicans* to quinalizarin at 4 and 8 µg/mL for 5 h. This effect was noticeable primarily at the higher concentrations of quinalizarin, as the level of gene expression decreased to 50%–70%, compared to the control (untreated cells) (Fig. 3).

### Induction of apoptosis and necrosis

**Fig 4:**
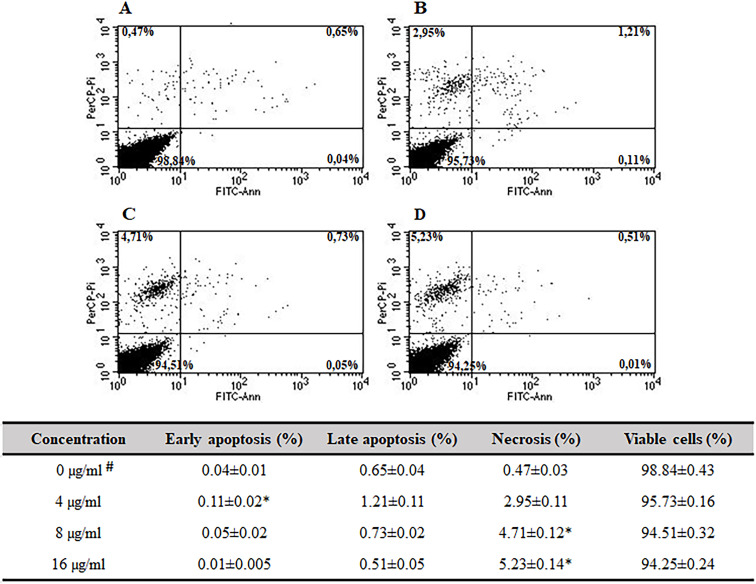
Effect of quinalizarin on early apoptosis, late apoptosis, and necrosis determined by Annexin V and propidium iodide staining. Control (untreated) cells (**A**); treatment with quinalizarin at 4 µg/mL (**B**), 8 µg/mL (**C**), and 16 µg/mL (**D**). Values are expressed as mean ± S.E. (Standard Error), %. #Untreated cells were used as a control. *Significant difference with the untreated group (*P* < 0.05).

FITC-Annexin V and propidium iodide (PI) staining were performed to determine the mode of the fungicidal activity of quinalizarin (through induction of apoptosis and/or necrosis). FITC-Annexin V is a sensitive probe with a high affinity for the membrane phospholipid phosphatidylserine (PS). PS is translocated from the inner to the outer leaflet of the plasma membrane in apoptotic cells. Staining with FITC-Annexin V is used in conjunction with propidium iodide, which is a vital dye. Viable cells with intact membranes exclude PI, whereas the membranes of dead and damaged cells are permeable to PI. Therefore, the staining patterns discriminate between live cells (Annexin V−/PI−) and early apoptosis (Annexin V+/PI−), necrosis (Annexin V+/PI+), and late apoptosis/necrosis (Annexin V+/PI+) (46). Apoptosis and necrosis were studied in *C. albicans* cells exposed to quinalizarin at 4, 8, and 16 µg/mL for 5 h. The total population of apoptotic cells was measured using flow cytometry (Fig. 4).

As presented above, the increase in the concentration of quinalizarin was accompanied by an increase in the number of necrotic cells, reaching 2.95% at 4 µg/mL (Fig. 4B), 4.71% at 8 µg/mL (Fig. 4C), and 5.23% at 16 µg/mL (Fig. 4D). Moreover, with the increasing concentration of quinalizarin, we observed a decrease in the number of late-apoptotic cells with a tendency to progress toward necrosis—from 1.21% at 4 µg/mL (Fig. 4B) and 0.73% at 8 µg/mL (Fig. 4C) to 0.51% at 16 µg/mL. In addition, there were slight changes in the early apoptosis phase, where cell loss and transition to necrosis were observed—from 0.11% at 4 µg/mL (Fig. 4B) to 0.01% at 16 µg/mL (Fig. 4D).

### Quinalizarin effects on other markers of apoptosis

**Fig 5:**
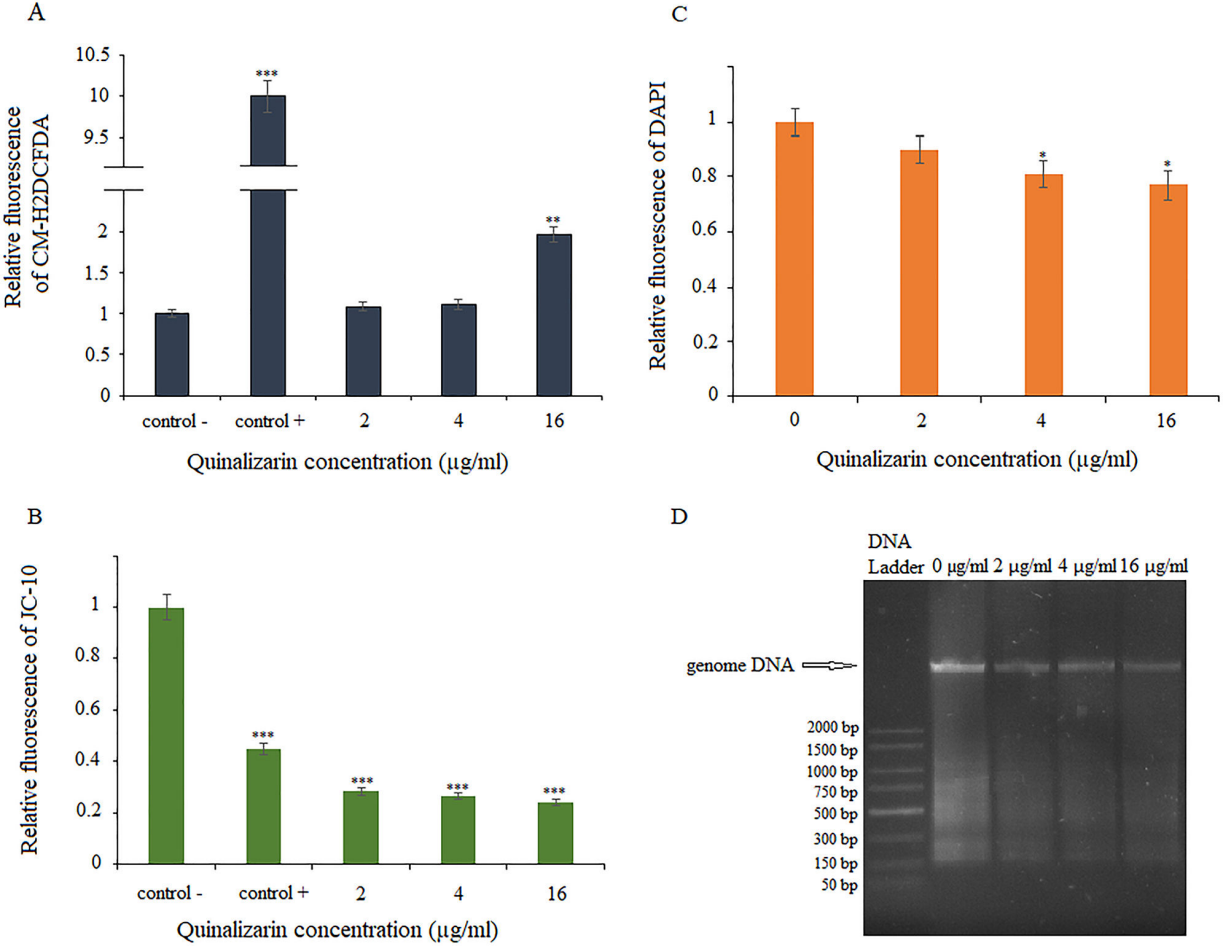
Effect of quinalizarin on various markers of apoptosis. ROS production determined by CM-H2DCFDA staining (**A**). 100 mM H_2_O_2_ was used as a positive control; mitochondrial membrane potential determined by JC-10 staining (**B**), 50 µM FCCP was used as a positive control; level of DNA loss demonstrated by DAPI staining (**C**); genomic DNA samples after electrophoresis on 1% agarose gel (**D**). Data are expressed as mean ± standard error of three independent experiments. *P < 0.05; *P < 0.01; and ***P < 0.001 [significance compared to the control (untreated cells) set at 1.0].

To examine the apoptotic effects of quinalizarin in greater detail, we first assayed ROS production, which is implicated in the induction and regulation of the apoptotic pathway in yeast. To check whether quinalizarin also affected the ROS levels in the *C. albicans* cells, the CM-H2DCFDA assay (chloromethyl derivative of H2DCFDA, an indicator of reactive oxygen species) was performed. The ROS levels were determined in the control (untreated) and quinalizarin-treated cells at three concentrations (4, 8, and 16 µg/mL). As shown in Fig. 5A, quinalizarin at the concentration of 16 µg/mL significantly increased the level of ROS in the *C. albicans* cells.

The dissipation of mitochondrial membrane potential is a key cellular phenomenon during early apoptosis, which leads to the opening of the transition pores of the mitochondrial membrane and the release of apoptogenic factors into the cytosol (47). We used the selective mitochondrial dye JC-10 to investigate mitochondrial function. In healthy cells with high MMP, JC-10 is concentrated and forms reversible red-fluorescent JC-10 aggregates (*λ*_ex_ = 540 nm/*λ*_em_ = 590 nm) in the mitochondria. In apoptotic or unhealthy cells, MMP collapse results in the failure to retain JC-10 in the mitochondria and a return of the dye to its monomeric green fluorescent form (*λ*_ex_ = 490 nm/*λ*_em_ = 525 nm). As shown in Fig. 5B, a significant loss of MMP was observed at all quinalizarin concentrations used, even at the subinhibitory concentrations of 2 and 4 µg/mL.

During the late stages of apoptosis, chromatin is damaged by the proteolysis of nuclear proteins, a process that results in DNA damage and chromatin condensation (48). To investigate the features of late apoptosis in response to quinalizarin, we used DAPI staining. DAPI binds to AT sites within the minor groove of DNA, where its fluorescence can be assessed. During apoptosis, the DNA content is reduced, resulting in less deep blue fluorescence. We observed insignificant differences in fluorescence between the quinalizarin-exposed cells and the untreated cells (control) (Fig. 5C). We confirmed these observations by genomic DNA analysis using agarose gel electrophoresis. *C. albicans* cells exposed to the various concentrations of quinalizarin did not show nuclear fragmentation associated with DNA damage (Fig. 5D).

### Effect of quinalizarin on human erythrocytes

**Fig 6:**
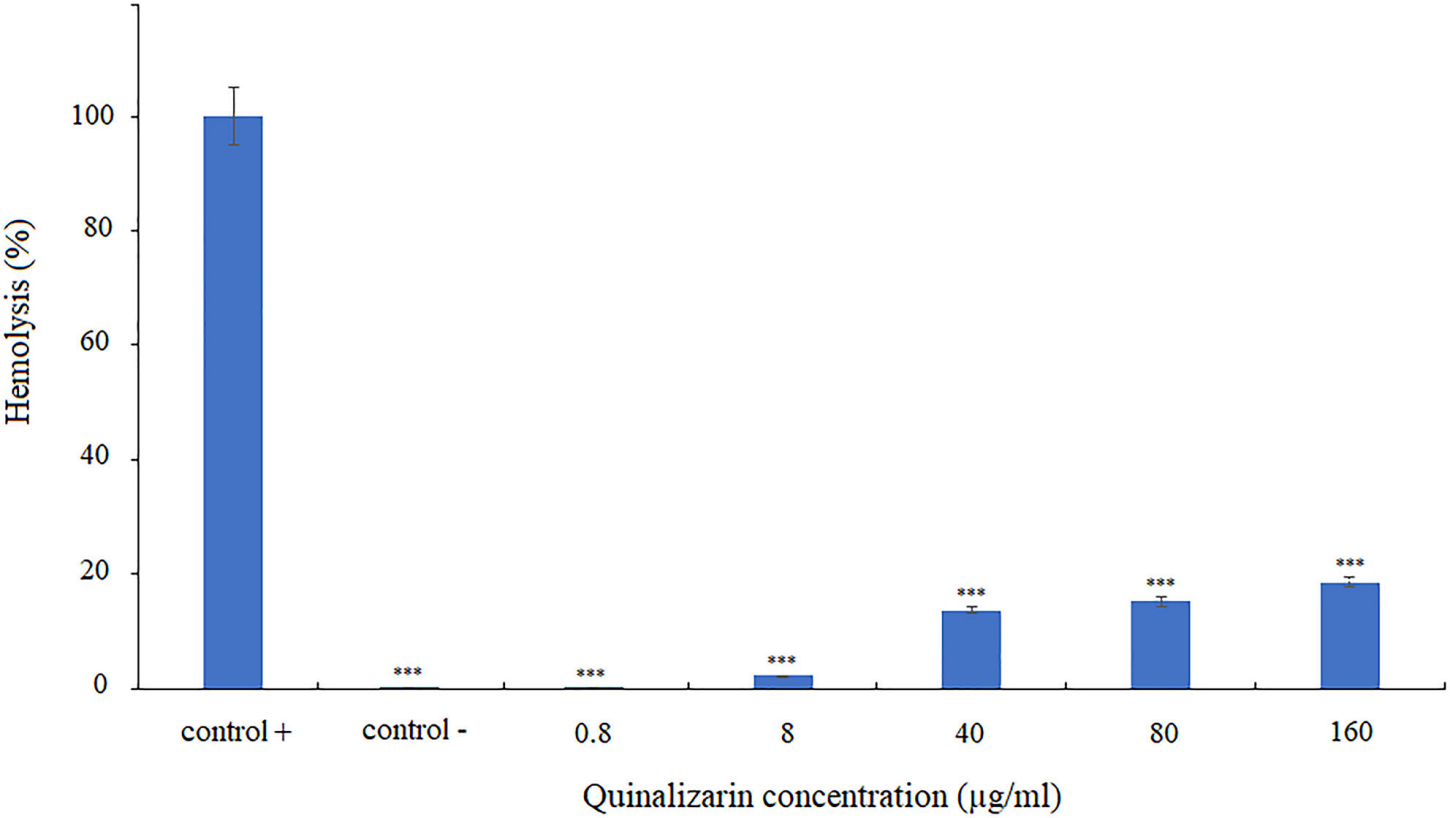
Effect of quinalizarin on human erythrocytes. Triton X-100 and PBS were used as positive and negative controls, respectively. Data are expressed as mean ± standard error of three independent experiments. ***P < 0.001 (significance compared to the positive control set at 100%).

In order to verify the effect of quinalizarin on healthy human cells, induction of erythrocyte lysis was assessed. To this end, human erythrocytes were incubated in the presence of different concentrations of quinalizarin (0.8–160 µg/mL). The results revealed that only high concentrations of the compound (40–160 µg/mL) significantly induced cell lysis, but at a level not exceeding 20% of erythrocytes. In turn, at a concentration corresponding to the MIC value, quinalizarin destroyed only 2.2% of erythrocytes (Fig. 6), which suggests that quinalizarin is safe for human blood cells at the effective antifungal concentration.

## DISCUSSION

In the present study, we investigated the effects of quinalizarin on the growth of pathogenic yeasts, which are often the causes of invasive fungal infections in patients with cancer. The selected yeast strains were used to determine the antifungal activity of quinalizarin, including reference *Candida* strains, fluconazole-sensitive and fluconazole-resistant clinical isolates of *C. albicans*, and one species of *Geotrichum* (Table 1). Quinalizarin showed fungistatic activity against all the tested species, including those resistant to fluconazole, which is most commonly used in the treatment of IFIs, even in 67.7% of cases (14). Our results place quinalizarin among compounds with antifungal activities, in addition to the already proven properties of this compound. In the light of the previous studies on anthraquinones, quinalizarin is as effective against *C. albicans* as the other representatives of this group, e.g., emodin (MIC = 12.5 µg/mL), aloe-emodin and its acetate (MIC = 6.1 µg/mL), purpurin (MIC = 1.28–5.12 µg/mL), and shikonin (MIC_80_ = 2–8 µg/mL) (49–52). The effectiveness of quinalizarin in the treatment of various types of cancer and our results suggesting its strong antifungal properties indicate the potential of this compound as a dual-activity drug in the treatment of cancer-associated candidiasis.

Subsequently, we determined the effect of quinalizarin on *C. albicans* pathogenicity factors. Our results show that quinalizarin is an effective inhibitor of both hyphal growth and cell aggregation in *Candida* (Fig. 1). In addition, our data demonstrate that quinalizarin has antibiofilm activity. Notably, this anthraquinone was able not only to inhibit biofilm formation but also to destroy the mature biofilm (Fig. 2). Biofilm formation is considered a virulence factor due to its ability to confer resistance to antifungal drugs and protect fungal cells from host immune responses (53, 54). Therefore, discovering new compounds with anti-biofilm properties seems to be crucial for the achievement of an effective therapy against *C. albicans*. Previously, it was reported that quinalizarin also reduced biofilm formation by Gram-positive bacterium, *Staphylococcus aureus* (55). It is generally believed that natural products, including anthraquinones, play an important role in the discovery and development of antibiofilm medicinal products (56). The hitherto exploration of new and effective natural compounds with antibiofilm effects was successful, and such compounds as *tt*-farnesol, epigallocatechin gallate, ellagic acid, cinnamaldehyde, carvacrol, xanthorrhizol, salvipisone, magnolol, honokiol, baicalein, quercetin, and emodin were identified (52, 57–59).

In order to gain more insight into the mechanism of quinalizarin activity as an inhibitor of *Candida* biofilm growth, we studied the transcriptional profile of nine genes related to hyphal formation and biofilm growth. Our results demonstrated that the treatment with quinalizarin at 8 µg/mL significantly decreased the levels of expression of nine genes associated with the formation of hyphae and biofilm (Fig. 3). The *EFG1* gene encoding the transcription factor positively regulates the expression of hypha-specific genes such as *ECE1* (60). *ECE1* is associated with both cell adhesion and hypha formation by the regulation of the extent of cell elongation (61). *BCR1* is also required for the adherence of cells and biofilm formation. Moreover, *BCR*1 is a regulator in the expression of several cell wall proteins necessary for adherence, for example, Als1, Als3, and Hwp1 (62, 63). Interestingly, our results indicate that adhesion-related genes *ALS1, ALS3*, and *HWP1* were also significantly downregulated following treatment with quinalizarin. The *CPH1, HYR1*, and *SAP4* genes were downregulated by quinalizarin as well. *CPH1* is a transcription factor involved in mating, filamentation on solid media, and pheromone-stimulated biofilms, *HYR1* is a hyphally regulated gene, and the *SAP4* gene encodes secretory aspartyl protease Sap4, which is involved in hypha formation and virulence (64–66).

In this study, we found that both cellular apoptosis and necrosis were the physiological mechanisms of *C. albicans* cell death induced by quinalizarin. We used multiple crucial markers of early and late apoptosis to conclusively implicate this process as a mechanism of quinalizarin-induced cell death. In our primary assay, apoptosis and necrosis were demonstrated by Annexin V and propidium iodide staining for phosphatidylyserine externalization and loss of membrane integrity, respectively. Apoptosis was investigated after 5 h of quinalizarin exposure at concentrations of 2, 4, and 16 µg/mL, and early apoptosis, late apoptosis, and necrosis were apparent in 0.11%–0.01%, 0.21%–0.51%, and 2.95%–5.23% of the cells, respectively. The data suggest that quinalizarin at concentrations below the MIC value causes *C. albicans* cells to undergo apoptosis, but a quinalizarin concentration increasing above the MIC value does not further induce programed cell death responses and induces necrosis (Fig. 4).

The early apoptosis was corroborated by ROS production and mitochondrial membrane potential assays. ROS and mitochondria play an essential role in apoptotic processes. During apoptosis, oxidative stress induces the mitochondrial permeability transition pore to open, which then triggers mitochondrial outer membrane permeabilization involved in the release of proapoptotic factors, leading to subsequent metacaspase activation (67). One of the physicochemical properties of anthraquinones is participation in redox reactions that generate deleterious reactive oxygen species resulting in oxidative stress (68). These features have been exploited in cancer therapy since the antitumor efficacy of chemotherapeutic drugs through enhancement of their cytotoxic effects has been proved. The intercellular ROS generation induced by antitumor drugs may trigger apoptosis in cancer cells (69–71). Previously, it has been demonstrated that the antitumor activity of quinalizarin may be related to these chemical properties. By generating ROS, quinalizarin inhibited the growth of melanoma cells (25), colorectal cancer cells (32), human breast cancer cells (34), and human esophageal cancer HCE-4 cells (35). On the other hand, ROS are toxic to *C. albicans*, and the induced production of endogenous ROS through oxidative damage causes apoptosis in yeast cells (72). Therefore, the use of certain specific antifungal agents may induce an apoptotic response of *C. albicans* by targeting and increasing the production of ROS, and this offers the possibility of controlling this pathogen. In this study, the quinalizarin-treated *C. albicans* cells exhibited elevated ROS levels and decreased mitochondrial membrane potential (Fig. 5).

The late apoptosis was confirmed by DNA damage and nuclear fragmentation assays (DAPI staining and agarose gel electrophoresis). The results showed a reduced amount of DNA in samples treated with quinalizarin at 2, 4, and 16 µg/mL for 5 h compared to the control (untreated cells). In addition, we used agarose gel electrophoresis as a method for detecting DNA fragments with a low molecular weight (180 bp or multiples thereof), corresponding to the size of individual nucleosomes or oligonucleosomes in DNA extracts (73). In our study, we did not detect DNA fragmentation in the genomic DNA samples subjected to electrophoresis (Fig. 5).

Since quinalizarin is highly toxic to cancer cells and pathogenic yeast cells, we investigated the effect of this compound on normal human cells. We used human erythrocytes and proved that quinalizarin was not toxic at the concentration corresponding to the MIC value (8 µg/mL); moreover, the level of hemolysis did not exceed 20% even at the high 20× MIC concentrations (160 µg/mL).

In conclusion, quinalizarin is effective and exhibits low toxicity in the treatment of *C. albicans* and other yeast infections. These results are preliminary and should be developed in the future, as they may contribute to the development of a new drug with dual activity in the treatment of cancer-associated candidiasis.
